# Unraveling the genetic complexities of combined retinal dystrophy and hearing impairment

**DOI:** 10.1007/s00439-021-02303-1

**Published:** 2021-06-20

**Authors:** Paulina Bahena, Narsis Daftarian, Reza Maroofian, Paola Linares, Daniel Villalobos, Mehraban Mirrahimi, Aboulfazl Rad, Julia Doll, Michaela A. H. Hofrichter, Asuman Koparir, Tabea Röder, Seungbin Han, Hamideh Sabbaghi, Hamid Ahmadieh, Hassan Behboudi, Cristina Villanueva-Mendoza, Vianney Cortés-Gonzalez, Rocio Zamora-Ortiz, Susanne Kohl, Laura Kuehlewein, Hossein Darvish, Elham Alehabib, Maria de la Luz Arenas-Sordo, Fatemeh Suri, Barbara Vona, Thomas Haaf

**Affiliations:** 1 Institute of Human Genetics, Julius Maximilians University Würzburg, 97074 Würzburg, Germany; 2 Ocular Tissue Engineering Research Center, Research Institute for Ophthalmology and Vision Science, Shahid Beheshti University of Medical Sciences, Tehran, Iran; 3 Department of Neuromuscular Disorders, UCL Queen Square Institute of Neurology, London, UK; 4 Universidad Nacional Autónoma de México, Mexico City, Mexico; 5 Department of Bioinformatics, University of Würzburg, Würzburg, Germany; 6 Department of Otolaryngology-Head and Neck Surgery, Tübingen Hearing Research Centre, Eberhard Karls University Tübingen, 72076 Tübingen, Germany; 7 Ophthalmic Epidemiology Research Center, Research Institute for Ophthalmology and Vision Science, Shahid Beheshti University of Medical Sciences, No. 23, Paidarfard St., Boostan 9 St., Pasdaran Ave., Tehran, 1666673111 Iran; 8 Amiralmomenin Hospital, Eye Research Center, Guilan University of Medical Sciences, Rasht, Iran; 9 Genetics Department, Asociación Para Evitar la Ceguera en México (APEC), Mexico City, Mexico; 10 Ophthalmic Department, Instituto de Seguridad y Servicios sociales de los Trabajadores del Estado, Hospital de Alta Especialidad, Puebla, Mexico; 11 Centre for Ophthalmology, Institute for Ophthalmic Research, Eberhard Karls University Tübingen, Tübingen, Germany; 12 University Eye Hospital, Centre for Ophthalmology, Eberhard Karls University Tübingen, Tübingen, Germany; 13 Faculty of Medicine, Neuroscience Research Center, Golestan University of Medical Sciences, Gorgan, Iran; 14 Student Research Committee, Department of Medical Genetics, School of Medicine, Shahid Beheshti University of Medical Sciences, Tehran, Iran; 15 Department of Genetics, National Institute of Rehabilitation Luis Guillermo Ibarra (INR), Mexico City, Mexico

## Abstract

Usher syndrome, the most prevalent cause of combined hereditary vision and hearing impairment, is clinically and genetically heterogeneous. Moreover, several conditions with phenotypes overlapping Usher syndrome have been described. This makes the molecular diagnosis of hereditary deaf–blindness challenging. Here, we performed exome sequencing and analysis on 7 Mexican and 52 Iranian probands with combined retinal degeneration and hearing impairment (without intellectual disability). Clinical assessment involved ophthalmological examination and hearing loss questionnaire. Usher syndrome, most frequently due to biallelic variants in *MYO7A* (USH1B in 16 probands), *USH2A* (17 probands), and *ADGRV1* (USH2C in 7 probands), was diagnosed in 44 of 59 (75%) unrelated probands. Almost half of the identified variants were novel. Nine of 59 (15%) probands displayed other genetic entities with dual sensory impairment, including Alström syndrome (3 patients), cone-rod dystrophy and hearing loss 1 (2 probands), and Heimler syndrome (1 patient). Unexpected findings included one proband each with Scheie syndrome, coenzyme Q10 deficiency, and pseudoxanthoma elasticum. In four probands, including three Usher cases, dual sensory impairment was either modified/aggravated or caused by variants in distinct genes associated with retinal degeneration and/or hearing loss. The overall diagnostic yield of whole exome analysis in our deaf–blind cohort was 92%. Two (3%) probands were partially solved and only 3 (5%) remained without any molecular diagnosis. In many cases, the molecular diagnosis is important to guide genetic counseling, to support prognostic outcomes and decisions with currently available and evolving treatment modalities.

**Supplementary Information:**

The online version contains supplementary material available at 10.1007/s00439-021-02303-1.

## Introduction

Neuro-sensory deficits are among the most prevalent congenital disorders in humans. Impaired hearing and/or vision can negatively affect a person's communication abilities, cognitive functions, and social competencies. Both senses work together and to some extent, one can help compensate for loss of the other. Therefore, deaf–blindness, including a wide range of hearing and vision levels (99% of patients have some residual hearing and/or vision capabilities), is more than the sum of hearing impairment (HI) and vision impairment (VI); it seriously impacts multiple areas of development. Children with dual sensory impairments have specific educational needs, requiring an interdisciplinary team of medical specialists and teachers (Dammeyer 2014).

Deaf–blindness may be acquired, e.g. by intrauterine infections (e.g. rubella, cytomegalovirus), premature birth, and cerebral palsy. However, in developed countries, the vast majority of congenital cases have a genetic basis. Usher syndrome (USH), a heterogeneous autosomal recessive disorder, accounts for approximately 50% of hereditary cases of combined deafness and blindness; it has an incidence of 1 in 6000–25,000 (Kimberling et al. 2010). In addition, variants in USH genes account for up to 10% of children diagnosed with non-syndromic HI (Jouret et al. 2019). The most severe form, USH1, is characterized by congenital severe to profound deafness, vestibular dysfunction, and retinal degeneration in the first decade of life. USH1 can be caused by variants in at least five genes (*MYO7A*, *USH1C*, *CDH23*, *PCDH15*, and *USH1G*). The most common form is USH2, accounting for diagnoses in two-thirds of USH patients, that is characterized by moderate to severe congenital HI and retinitis pigmentosa (RP) in the second decade of life. USH2 results from mutations in at least three genes (*USH2A*, *ADGRV1*, and *WHRN*); mutations in *USH2A* are by far the most common cause accounting for 90% of USH2 cases (Bonnet et al. 2016). The mildest form, USH3, exhibits variable, usually progressive, HI and RP later in life and has been associated with variants in *CLRN1* (Hereditary Hearing Loss Homepage; https://hereditaryhearingloss.org; Retinal Information Network; https://sph.uth.edu/retnet/disease.htm).

Here, we performed whole exome analysis (WEA) of 59 unrelated, mainly consanguineous families with at least one proband clinically diagnosed with HI and retinal degeneration. Our study highlights the enormous molecular genetic heterogeneity of combined HI and VI and the need to analyze larger gene panels (for both diseases of the auditory and visual system) in addition to known or suspected USH genes.

## Materials and methods

### Patients

This study was approved by the ethics committees at the Medical Faculty of Würzburg University, Germany (approval number 46/15), the National Institute of Rehabilitation Luis Guillermo Ibarra (INR), Mexico (no. 12/13), and the Shahid Beheshti University of Medical Sciences, Tehran, Iran and was carried out following the ethical principles of the declaration of Helsinki. Written informed consent was obtained from all participating families prior to their inclusion in the study.

We recruited 59 unrelated probands with combined HI and retinal degeneration resulting in reduced vision and nyctalopia. None of the probands reportedly suffered from intellectual disability. Fifty-two Iranian index patients from consanguineous families were seen by ophthalmologists in different centers and were cataloged in the Iranian Inherited Retinal Disease Registry (NCT04131400) (Sabbaghi et al. 2020). Seven non-consanguineous Mexican probands were recruited by medical geneticists in the Asociación Para Evitar la Ceguera en México (APEC) and the National Institute of Rehabilitation (INR), Mexico City.

Medical, drug and familial histories were recorded. Ocular examinations (Supplementary Table 1) of all Iranian patients were performed at Labbafinejad Medical Center, affiliated with the Shahid Beheshti University of Medical Sciences. These included examination of the best corrected visual acuity (VA) using Snellen charts, slit lamp biomicroscopy of the anterior and posterior segments (using + 90 lens), Goldmann tonometry, and color vision and visual field testing using a Humphrey Field Analyzer II, model 750 (Zeiss Humphrey Systems, Dublin, CA, USA). For patients with VA scores under 20/200, a semi-quantitative scale was used, including counting fingers at different distances, hand movements and light perception. In many cases, depending on the level of vision, standard full field electroretinography (ERG) was performed under photopic and scotopic conditions. Ocular imaging included color fundus photography (CFP), spectral domain optical coherence tomography (OCT) (6 mm scans centered from the fovea, using a Spectralis imaging platform, Heidelberg, Germany), and fundus autofluorescence (FAF) imaging (HRA, Heidelberg, Germany). Patients with apparent USH were classified with mild, moderate or severe cystoid macular edema (CME), using the grading system of Sliesoraityte et al. (2015). The following features were considered: subretinal fluid without clearly detectable cystic macular lesion (CML) boundaries, central macular thickness, largest diameter of CML, calculated mean of all detectable CMLs, total number of detectable CML, and retinal layers affected by CML.

HI was determined by anamnesis and by questionnaire. Audiograms were available from probands 1, 6, 8, 9, 12, 24, 25, 42, 44, 47, 52, 54, and 58 (Supplementary Table 1). Severity of HI was assessed by averaging pure-tone thresholds of the better hearing ear over 0.5, 1, 2, and 4 kHz. Averaged thresholds between 41–70 dB represent moderate, 71–95 dB severe, and > 95 dB profound HL. Interpretation of audiometry followed the recommendations of the GENDEAF study group (Mazzoli et al. 2003).

### Sequence analysis and variant classification

Genomic DNAs of probands and available family members were extracted from whole blood samples using a standard salting out method and quantified using Qubit 2.0 (Life Technologies, Carlsbad, CA, USA). Exome capture was performed using the TruSeq Rapid Exome or Nextera DNA Exome (Illumina, San Diego, CA, USA) enrichment according to manufacturer’s protocols. Libraries were paired-end sequenced (2 × 76 bp) with the v2 reagent kit (Illumina) on a NextSeq 500 (Illumina) sequencer. The generated sequences were de-multiplexed and mapped to the human genome reference (NCBI build 37/hg19 version) with Burrows Wheeler Aligner.

First, a targeted analysis of the following USH-associated genes was performed using GensearchNGS software (PhenoSystems SA, Wallonia, Belgium): *ABDH12* (OMIM 613599, NM_001042472.2), *ADGRV1* (OMIM 602851, NM_032119.3), *CDH23* (OMIM 605516, NM_022124.5), *CEP250* (OMIM 609689, NM_007186.5), *CLRN1* (OMIM 606397, NM_001195794.1), *MYO7A* (OMIM 276903, NM_000260.3), *PCDH15* (OMIM 605514, NM_001142763.1), *PDZD7* (OMIM 612971, NM_001195263.1), *USH1C* (OMIM 605242, NM_153676.3), *USH1G* (OMIM 607696, NM_173477.4), *USH2A* (OMIM 608400, NM_206933.2), and *WHRN* (OMIM 607928, NM_015404.3). In a second step, the whole exome was analyzed in patients without molecular USH diagnoses. A refuted USH1J gene, *CIB2* (Riazuddin et al. 2012; Booth et al. 2018), a questionable USH1M gene, *ESPN* (Ahmed et al. 2018), a refuted USH3B gene, *HARS* (Puffenberger et al. 2012; DiStefano et al. 2019), and a recently identified USH4 gene, *ARSG* (Peter et al. 2021), were not included in our original screen, but rather in exome-wide analysis.

Single nucleotide variants (SNVs) and small indels (< 15 bp) were analyzed using GensearchNGS, MutationDistiller (Hombach et al. 2019), and Moon Diploid (http://www.diploid.com/moon). Alternative alleles present at > 20% and a minor allele frequency (MAF) < 0.01 were used for variant filtering. Population-specific allele frequencies were assessed using gnomAD (Karczewski et al. 2020) and the Greater Middle Eastern (GME) variome (Scott et al. 2016). PolyPhen-2 (Adzhubei et al. 2010), MutationTaster (Ng and Henikoff 2001), and SIFT (Schwarz et al. 2014) were used to analyze pathogenicity of SNVs. Variants were queried in the Deafness Variation Database (DVD) (Azaiez et al. 2018), the Human Gene Mutation Database (HGMD) (Stenson et al. 2014), and the Leiden Open Variation Database (LOVD) (https://www.lovd.nl). Potential splicing effects of variants were classified by in silico prediction tools such as SpliceSiteFinder-like (Shapiro and Senapathy 1987), MaxEntScan (Yeo and Burge 2004), NNSPLICE (Reese et al. 1997), Genesplicer (Pertea et al. 2001), and Human Splicing Finder (Desmet et al. 2009). Variants were classified according to the recommendations of the American College of Medical Genetics and Genomics (ACMG) and the Association for Molecular Pathology (AMP) (Richards et al. 2015), as well as established recommendations for hereditary HI (Oza et al. 2018). Potentially disease-causing and compound heterozygous variants were validated by segregation analysis in the index patients and available family members using Sanger sequencing on an ABI 3130xl 16-capillary sequencer (Life Technologies). Primer sequences were designed using Primer 3 (https://primer3.org).

In probands with a single pathogenic *USH2A* variant (50, 51, and 57), partially solved (45 and 53), and unsolved cases (55, 57, and 59), multiplex ligation-dependent probe amplification (MLPA) with the SALSA Probemix P361, P362, and P292 (MRC Holland, Amsterdam, The Netherlands) was used to exclude copy number variations (CNVs) in *USH2A* and *PCDH15*. In addition, these probands were screened by Sanger sequencing for deep intronic mutations (DIMs) in *USH2A* introns 27 (c.5573-843A>G), 40 (c.7595-2144A>G), 44 (c.8845 + 628C>T), 50 (c.9959-4159A>G), and 64 (c.14134-3169A>G) (Vache et al. 2012; Liquori et al. 2016; Baux et al. 2017). In some probands showing multi-locus variation (42, 43, 44, 45, and 58), the Infinium Global Screening Array-24 v1.0 BeadChip (Illumina, SanDiego, CA, USA) was applied for genome-wide CNV screening.

### In vitro splice assays

To evaluate the impact of the *ADGRV1* c.9623 + 3A>C and *PDSS2* c.702 + 1G>A splice variants, PCR amplification from patient and control genomic DNA ensued following a modified protocol (Tompson and Young 2017). For testing of the *ADGRV1* variant, amplification of a region including exon 44 (176 bp) with an additional 589 bp (5′) and 340 bp (3′) from the flanking intronic region was performed using primers containing *Xho*I and *Bam*HI restriction sites (forward primer with a *Xho*I restriction site: 5′-aattctcgagTTTGCTGGTTCTTGAGCTTC-3′ and reverse primer with a *Bam*HI restriction site: 5′-attggatccTCATTCACTTGGTTTGAGCAG-3′). Similarly, for testing of the *PDSS2* variant, amplification of a region containing exon 4 (71 bp) that included 390 bp (5′) and 1380 bp (3′) from the flanking intronic regions was performed (forward primer with a *Xho*I restriction site: 5′-aattctcgagTTGTAATTGCTCCCAGAATGG-3′ and reverse primer with a *Bam*HI restriction site: 5′-attggatccTGACTTCAAATCCCTGAGAGC-3′). Following PCR amplification and clean-up, restriction enzyme digestion of the PCR fragment and pSPL3 exon trapping vector was performed prior to ligation between exons A and B of the linearized pSPL3-vector. The vector was transformed into DH5α competent cells (NEB 5-alpha, New England Biolabs, Frankfurt am Main, Germany), plated, and incubated overnight. The wild-type and mutant-containing vector sequences were Sanger sequence confirmed. Vectors containing mutant and wild-type sequence were transfected into HEK 293T cells (ATCC) at a density of 2 × 10^5^ cells per milliliter. 1 µg of the respective pSPL3 vectors were transiently transfected using 3 µl of FuGENE 6 Transfection Reagent (Promega, Walldorf, Germany). An empty vector and transfection negative reactions were included as controls. The transfected cells were harvested 24 h after transfection. Total RNA was prepared using miRNAeasy Mini Kit (Qiagen, Hilden, Germany). Approximately 1 µg of RNA was reverse transcribed using a High Capacity RNA-to-cDNA Kit (Applied Biosystems, Foster City, CA, USA) following the manufacturer’s protocols. The cDNA was PCR amplified using vector-specific SD6 forward (5′-TCTGAGTCACCTGGACAACC-3′) and SA2 reverse (5′-ATCTCAGTGGTATTTGTGAGC-3′) primers. The amplified fragments were visualized on a 2% agarose gel and Sanger sequenced. *ADGRV1* fragments were cloned using the TA cloning dual promoter with pCRII kit (Invitrogen, Karlsruhe, Germany) and subsequently Sanger sequenced.

## Results

### Clinical and molecular characterization of the analyzed cohort

Staged exome diagnostics was performed on 59 unrelated probands with co-occurring HI and VI (due to retinal dystrophy) and without apparent intellectual disability. Patient age at the time of recruitment ranged from 8 to 65 years with a mean age of 34 years. Clinical parameters are presented in Supplementary Table 1. The reported visual acuity ranged from 20/30 Snellen equivalent to no light perception. The most common symptom was nyctalopia accompanied by bilateral reduced vision. The most common ophthalmological findings included waxy pallor discs, retinal atrophy, mottling and diffuse bone spicule pigmentary changes, cataracts, and macular edema. With exception of proband 11, intraocular pressure of both eyes by Goldmann tonometry was normal. The majority (64%) of the probands presented with prelingual HI, the rest with postlingual HI. If available, tympanograms showed normal middle ear function.

**Fig. 1:**
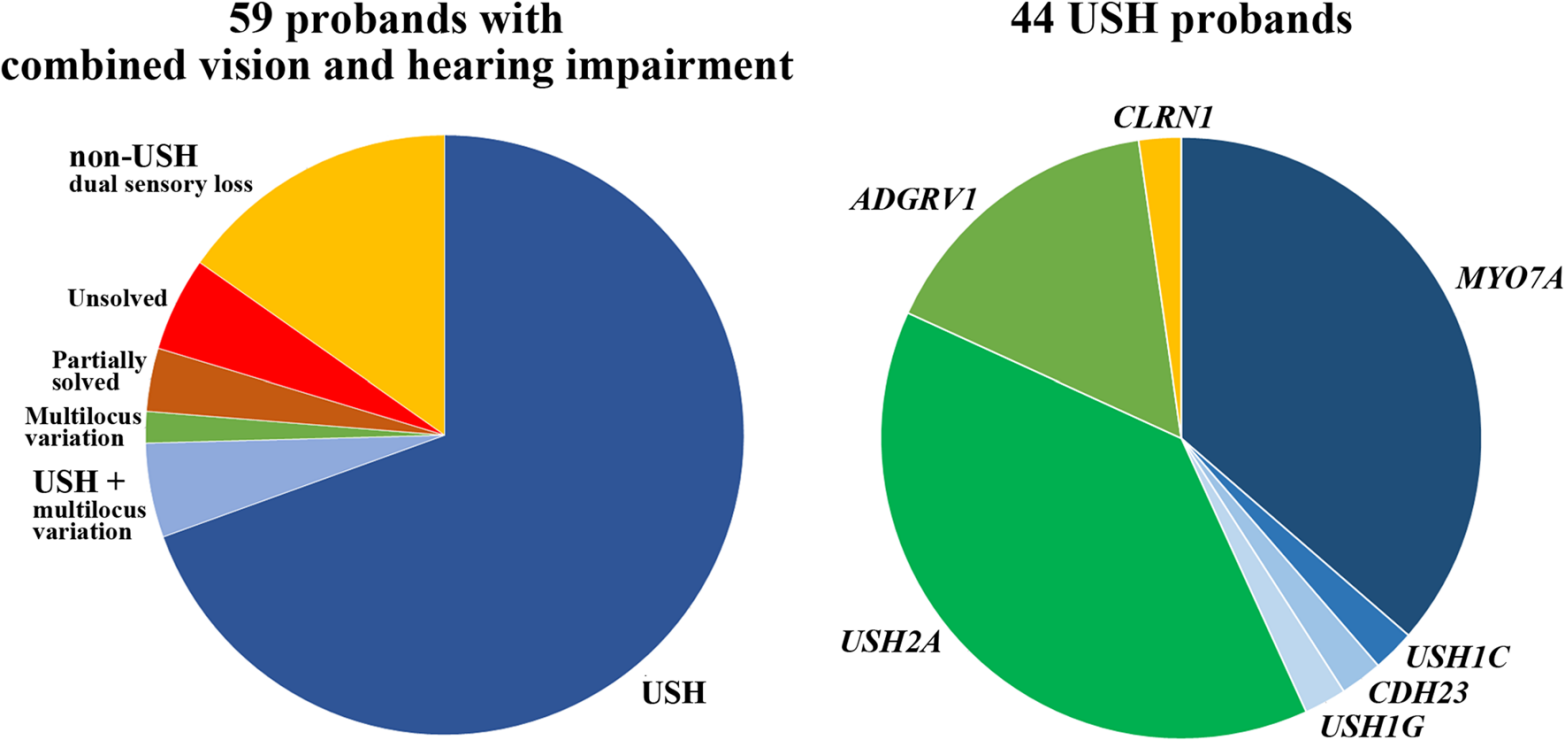
The left diagram shows the diagnostic yield in 59 probands with combined vision and hearing impairment. As expected, USH was the most common diagnosis accounting for 75% of cases. Please note that three USH (+ multi-locus variation) probands exhibited variants in additional genes for HI and VI. Different non-USH syndromes with co-occurring HI and VI were found in 15% of cases. The right diagram represents the mutational spectrum in the 44 USH probands. Mutations in USH1 genes are marked in different shades of blue and in USH2 genes in green

Forty-seven of the 52 (90%) cases from consanguineous Iranian families and all 7 cases from non-consanguineous Mexican families were solved. The overall diagnostic yield was 92% (54 of 59). A molecular diagnosis pointing to USH accounted for 44 of 59 (75%) and non-USH syndromes for 9 of 59 (15%) index probands. In three of the USH cases, additional variants in genes for non-syndromic HI (*OTOG, TECTA*) or *ABCA4*-related VI (Stargardt disease, cone-rod dystrophy, and RP) may modify/aggravate the phenotype (Fig. 1, left). In one of 59 (2%) probands, HI and VI were caused by variants in different genes. Two additional probands with a blended phenotype were partially solved.

### Usher syndrome as the most common cause of dual sensory loss

**Fig. 2:**
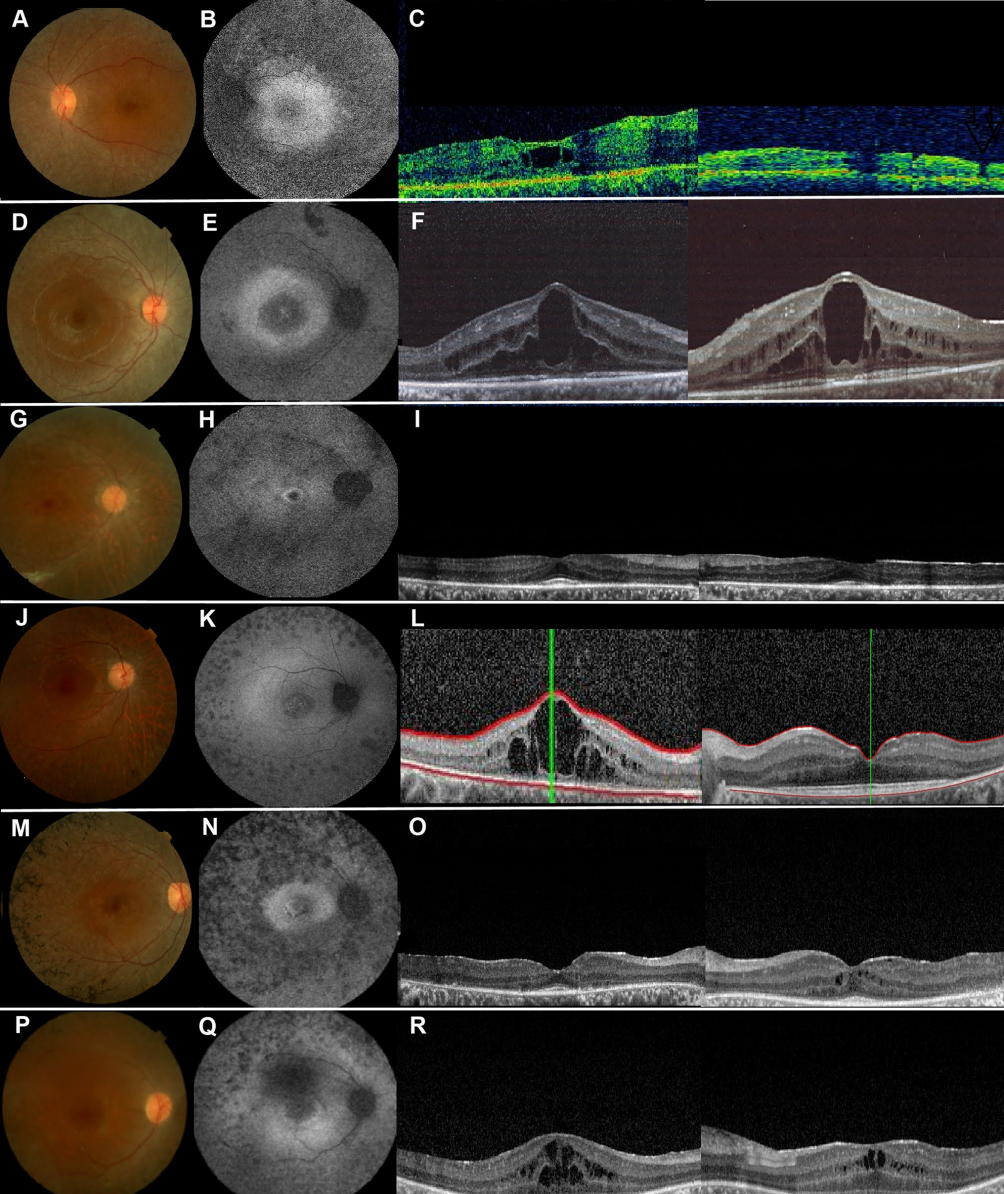
Retinal phenotype of selected probands with co-occurring HI and VI due to USH entities consistent with retinitis pigmentosa. **A**, **D**, **G**, **J**, **M**, **P** Fundus photography (FP), **B**, **E**, **H**, **K**, **N**, **Q** fundus autofluorescence (FAF), and **C**, **F**, **I**, **L**, **O**, **R** optical coherence tomography (OCT) imaging of patients 1 (**A**–**C**) and 7 (**D**–**F**) with USH1B, patient 18 (**G**–**I**) with USH1C, patient 41 (**J**–**L**) with USH1D, patient 21 (**M**–**O**) with USH2A, and patient 37 (**P**–**R**) with USH3A. Note retinal atrophy along the arcades in all patients, and bone spicules in patient 21 on FP imaging, decreased autofluorescence along the arcades, and increased and/or decreased autofluorescence centrally in all patients on FAF imaging, and macular pathology on OCT imaging in all patients, i.e. atrophy in patient 1, cystoid macular edema in patients 1, 7, 41, 21, and 37, and epiretinal membrane in patient 18

Forty-four patients showed biallelic variants in known USH genes, approximately half of the variants being novel (Table 1; Fig. 1, right). Nineteen of these 44 patients suffered from USH1, 24 from USH2, and only one from USH3. The most frequently affected genes in our cohort were *MYO7A* (USH1B) in 16 patients and *USH2A* (USH2A) in 17 patients, followed by *ADGRV1* (USH2C) in 7 patients. Biallelic variants in *USH1C*, *CDH23* (USH1D), *USH1G*, and *CLRN1* (USH3A) were diagnosed in single patients each. It is noteworthy that although the vast majority of our USH probands were from consanguineous families, compound heterozygous variants were found in 18 of 44 (41%) cases. Fundus examination, FAF, and OCT of selected patients with USH showed findings consistent with RP (Fig. 2). Using a grading system for CME (Sliesoraityte et al. 2015), moderate and severe cystic lesions were most prevalent among patients with USH1B and biallelic *MYO7A* variants (Supplementary Table 1). Bilateral pantonal profound sensorineural HI without vestibular dysfunction was the most common form of HI in the USH cohort (Supplementary Table 1).

**Table 1:** Biallelic variants and genotypes in USH genes

ID	Gene	Variant	Protein	Zygosity^a^	Classification	References	Phenotype
1	*MYO7A*	c.1969C>T	p.(Arg657Trp)	Hom	Pathogenic	Cremers et al. (2007)	USH1B
2	*MYO7A*	c.73G>A	p.(Gly25Arg)	Hom	Pathogenic	Watanabe et al. (2008)	USH1B
3	*MYO7A*	c.5617C>T	p.(Arg1873Trp)	Het	Pathogenic	Roux et al. (2006)	USH1B
	*MYO7A*	c.2904G>T	p.(Glu968Asp)	Het	Pathogenic	Bonnet et al. (2016)	
4	*MYO7A*	c.5573T>C	p.(Leu1858Pro)	Hom	Pathogenic	Bademci et al. (2016)	USH1B
5	*MYO7A*	c.397dup	p.(His133Profs*7)	Hom	Pathogenic	Bonnet et al. (2011)	USH1B
6	*MYO7A*	c.2282 + 1G>C	p.?	Het	Pathogenic	Novel	USH1B
	*MYO7A*	c.721C>T	p.(Arg241Cys)	Het	Pathogenic	Bharadwaj et al. (2000)	
7	*MYO7A*	c.397dup	p.(His133Profs*7)	Het	Pathogenic	Bonnet et al. (2011)	USH1B
	*MYO7A*	c.4513G>T	p.(Glu1505*)	Het	Pathogenic	Kooshavar et al. (2018)	
8	*MYO7A*	c.487G>A	p.(Gly163Arg)	Hom	Pathogenic	Roux et al. (2006)	USH1B
9	*MYO7A*	c.3564_3570delinsA	p.(Tyr1188*)	Hom	Pathogenic	Duzkale et al. (2013)	USH1B
10	*MYO7A*	c.6204dup	p.(Ile2069Tyrfs*7)	Het	Pathogenic	Cremers et al. (2007)	USH1B
	*MYO7A*	c.3564_3570delinsA	p.(Tyr1188*)	Het	Pathogenic	Duzkale et al. (2013)	
11	*MYO7A*	c.722G>A	p.(Arg241His)	Het	Likely pathogenic	Bademci et al. (2016)	USH1B
	*MYO7A*	c.1388A>G	p.(Gln463Arg)	Het	Likely pathogenic	Novel	
12	*MYO7A*	c.75_82del	p.(Ala26Glufs*13)	Hom	Pathogenic	Novel	USH1B
13	*MYO7A*	c.5510T>C	p.(Leu1837Pro)	Het	Likely pathogenic	Jiang et al. (2015)	USH1B
	*MYO7A*	c.487G>A	p.(Gly163Arg)	Het	Pathogenic	Sloan-Heggen et al. (2016)	
14	*MYO7A*	c.496del	p.(Glu166Argfs*5)	Het	Pathogenic	Riazuddin et al. (2008)	USH1B
	*MYO7A*	c.4117C>T	p.(Arg1373*)	Het	Pathogenic	Jaijo et al. (2006)	
15	*MYO7A*	c.6228_6232del	p.(Asp2076Glufs*50)	Hom	Pathogenic	Novel	USH1B
16	*MYO7A*	c.2914C>T	p.(Arg972*)	Hom	Pathogenic	Riazuddin et al. (2008)	USH1B
18	*USH1C*	c.2191C>T	p.(Arg731Trp)	Het	Uncertain significance	Novel	USH1C
	*USH1C*	c.658C>G	p.(Arg220Gly)	Het	Uncertain significance	Novel	
41	*CDH23*	c.7921G>A	p.(Asp2641Asn)	Hom	Likely pathogenic	Novel	USH1D
17	*USH1G*	c.742C>T	p.(Gln248*)	Hom	Pathogenic	Bonnet et al. (2016)	USH1G
19	*USH2A*	c.4732C>T	p.(Arg1578Cys)	Hom	Likely pathogenic	Le Quesne Stabej et al. (2012)	USH2A
20	*USH2A*	c.236_239dup	p.(Gln81Tyrfs*28)	Hom	Pathogenic	Khalaileh et al. (2018)	USH2A
21	*USH2A*	c.1571C>T	p.(Ala524Val)	Het	Likely pathogenic	Novel	USH2A
	*USH2A*	c.4628-2A>T	p.?	Het	Pathogenic	Novel	
22	*USH2A*	c.11955G>C	p.(Trp3985Cys)	Hom	Likely pathogenic	Novel	USH2A
23	*USH2A*	c.12394del	p.(Leu4132Trpfs*35)	Hom	Pathogenic	Sloan-Heggen et al. (2015)	USH2A
24	*USH2A*	c.11357del	p.(Pro3786Leufs*6)	Hom	Pathogenic	Novel	USH2A
25	*USH2A*	c.13510G>T	p.(Glu4504*)	Het	Pathogenic	Gao et al. (2021)	USH2A
	*USH2A*	c.13018G>C	p.(Gly4340Arg)	Het	Likely pathogenic	Bonnet et al. (2016)	
26	*USH2A*	c.12067-2A>G	p.?	Hom	Pathogenic	Auslender et al. (2008)	USH2A
27	*USH2A*	c.2512C>T	p.(Gln838*)	Het	Pathogenic	Novel	USH2A
	*USH2A*	c.2299del	p.(Glu767Serfs*21)	Het	Pathogenic	Eudy et al. (1998)	
28	*USH2A*	c.5251_5267del	p.(Gly1751Leufs*2)	Het	Pathogenic	Novel	USH2A
	*USH2A*	c.8141G>A	p.(Trp2714*)	Het	Pathogenic	Baux et al. (2014)	
29	*USH2A*	c.5521G>A	p.(Gly1841Arg)	Het	Likely pathogenic	Novel	USH2A
	*USH2A*	c.7915T>C	p.(Ser2639Pro)	Het	Likely pathogenic	Bonnet et al. (2016)	
30	*USH2A*	c.8497dup	p.(Ser2833Lysfs*2)	Hom	Pathogenic	Novel	USH2A
31	*USH2A*	c.12067-1G>C	p.?	Hom	Pathogenic	Novel	USH2A
32	*USH2A*	c.8682-1G>A	p.?	Het	Pathogenic	Novel	USH2A
	*USH2A*	c.2014C>T	p.(Gln672*)	Het	Pathogenic	Pierrache et al. (2016)	
42	*USH2A*	c.11389 + 3A>T	p.?	Hom	Pathogenic	Soens et al. (2017)	USH2A
	*OTOG*	c.7454del	p.(Arg2485Hisfs*77)	Hom	Pathogenic	Downie et al. (2020)	DFNB18B
	*PRPF31*	c.632G>A	p.(Arg211Gln)	Het	Uncertain significance	Novel	RP11, dominant
	*ROM1*	c.859C>T	p.(Arg287Trp)	Het	Uncertain significance	Novel	RP7, digenic
43	*USH2A*	c.5438C>A	p.(Ser1813*)	Het	Pathogenic	Novel	USH2A
	*USH2A*	c.7595-2144A>G	p.? (DIM)	Het	Pathogenic	Liquori et al. (2016)	
	*TECTA*	c.2572G>A	p.(Asp858Asn)	Het	Uncertain significance	Novel	DFNA8/12
58	*USH2A*	c.236_239dup	p.(Gln81Tyrfs*28)	Hom	Pathogenic	Khalaileh et al. (2018)	USH2A
	*KCNQ1*	c.733_734del	p.(Gly245Argfs*39)	Het	Pathogenic	Amirian et al. (2018)	Long QT 1
33	*ADGRV1*	c.9623 + 3A>C^b^	p.?	Hom	Likely pathogenic	Novel	USH2C
34	*ADGRV1*	c.15736C>T	p.(Arg5246*)	Hom	Pathogenic	Oishi et al. (2014)	USH2C
35	*ADGRV1*	c.4231del	p.(Ala1411Profs*6)	Hom	Pathogenic	Novel	USH2C
36	*ADGRV1*	c.4231del	p.(Ala1411Profs*6)	Het	Pathogenic	Novel	USH2C
	*ADGRV1*	c.10088_10091del	p.(Val3363Aspfs*11)	Het	Pathogenic	Ebermann et al. (2009)	
38	*ADGRV1*	c.9512T>C	p.(Leu3171Ser)	Hom	Uncertain significance	Novel	USH2C
52	*ADGRV1*	c.2261T>C	p.(Val754Ala)	Het	Uncertain significance	Myers et al. (2018)	USH2C
	*ADGRV1*	c.10878A>C	p.(Lys3626Asn)	Het	Uncertain significance	Novel	
	*ABCA4*	c.4919G>A	p.(Arg1640Gln)	Hom	Pathogenic	Simonelli et al. (2000)	Stargardt 1
56	*ADGRV1*	c.5167C>G	p.(Pro1723Ala)	Het	Uncertain significance	Novel	USH2C
	*ADGRV1*	c.14939T>C	p.(Val4980Ala)	Het	Uncertain significance	Novel	
37	*CLRN1*	c.630del	p.(Phe210Leufs*5)	Hom	Pathogenic	Novel	USH3A
	*CLRN1*	c.625T>A	p.(Phe209Ile)	Hom	Uncertain significance	Novel	USH3A

^a^Hom = homozygous; Het = heterozygous

^b^Testing with an in vitro splice assay showed a mixture of amplicons, r.9495_9623del p.(Tyr3166_Arg3208del), r.9530_9623del p.(Gly3177Glufs*5), and r.9448_9623del p.(Ala3150Serfs*11)

Patient 33 displayed a homozygous likely pathogenic splice variant, *ADGRV1* c.9623 + 3A>C (Table 1). In vitro splice assays showed three bands after RT-PCR (Supplementary Fig. 1). TA cloning showed 54% of transcripts using a first cryptic splice site 129 nucleotides before the native exon 44 splice donor site, leading to an in-frame deletion, r.9495_9623del p.(Tyr3166_Arg3208del); 38% of transcripts where splicing involved the second cryptic splice donor site 94 nucleotides before the exon 44 native splice site, causing a frameshift, r.9530_9623del p.(Gly3177Glufs*5), and 8% of misspliced transcripts showed skipping of exon 44, r.9448_9623del p.(Ala3150Serfs*11), also resulting in a frameshift. The wild-type minigene showed two main bands comprised of an upper band containing the expected exon 44 sequence (433 bp) and a lower band showing a skipped exon 44 (257 bp) that maps to exon 1 of a shorter transcript (ENST00000640464.1) lacking a splice acceptor site for splicing.

No pathogenic variants in *CIB2* (USH1J), *ESPN* (USH1M), *HARS* (USH3B), and *ARSG* (USH4) were found by WEA in the remaining 15 non-USH probands. Probands 50, 51, and 57 who exhibited single variants in *USH2A*, were analyzed by MLPA and Sanger sequencing to exclude CNVs and DIMs, which are not detectable by WEA. In addition to a novel pathogenic variant, c.5438C>A p.(Ser1813*), proband 43 was endowed with a well-known DIM, c.7595-2144A>G, in exon 40 (Table 1). Since her unaffected sister displayed only the pathogenic nonsense variant but not the DIM, this is consistent with the diagnosis USH2A in the index patient. Moreover, proband 43 (but not her unaffected sister) displayed a variant of uncertain significance (VUS) in *TECTA* (Table 1), which is associated with autosomal dominant deafness (DFNA8/12) and may contribute to her HI.

Proband 42, a 25-year-old female, with bilateral severe prelingual HI, bilateral reduced vision and nyctalopia starting at the age of 7 years, macular atrophy, diffuse retinal atrophy and pigmentary changes displayed a homozygous splice mutation, *USH2A* c.11389 + 3A>T (Table 1), which has been associated with retinal dystrophy and USH2A. An in vitro minigene assay has been reported, confirming truncation of 48 bp due to usage of a cryptic donor splice site in exon 58 (Soens et al. 2017). In addition, proband 42 was endowed with a homozygous missense variant, c.7454del p.(Arg2485Hisfs*77) in *OTOG* (Table 1), which causes autosomal recessive deafness (DFNB18B; OMIM 614945) (Downie et al. 2020) and may aggravate the HI phenotype. Her father and two unaffected siblings were heterozygous for this variant. The VI of proband 42 may be modified by the heterozygous variant c.632G>A p.(Arg211Gln) in *PRPF31* (Table 1), which has been associated with RP11 (OMIM 600138) in a single sporadic RP case (Rose et al. 2016). Although the MAF of this variant is about 0.001, functional studies have associated incomplete penetrance and variable expressivity of RP11 with *PRPF31* mRNA levels (Rivolta et al. 2006; Rio Frio et al. 2009). Moreover, proband 42 displayed a heterozygous VUS, c.632G>A p.(Arg287Trp) in *ROM1* (Table 1), which can cause RP7 together with mutations in *PRPH2* (Kajiwara et al. 1994). In this example of digenic RP, *ROM1* is considered as a dominant modifier of *PRPH2* (Nadeau 2001).

**Fig. 3:**
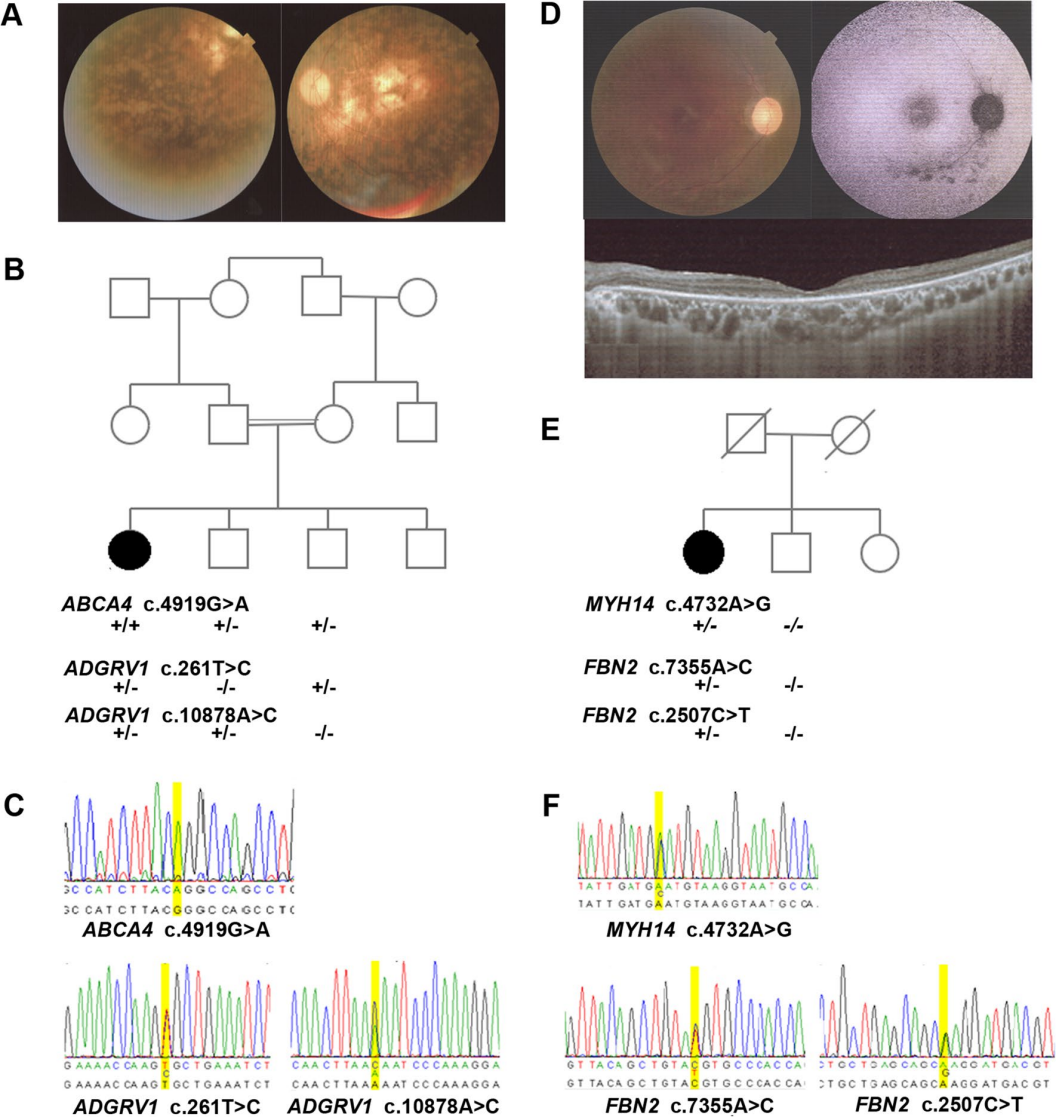
Possible coincidence and genetic heterogeneity underlying combined HI and VI. **A** Fundus photography (FP) of proband 52 showing central retinal atrophy and pigment clumping in both eyes consistent with *ABCA4*-spectrum disease. **B**, **C** Pedigree and segregation analysis of disease-causing variants in *ABCA4* and *ADGRV1*. **D** FP, fundus autofluorescence (FAF) and optical coherence tomography (OCT) imaging of proband 44 showing decreased autofluorescence along the inferior arcade and centrally on FAF imaging (right eye) and retinal atrophy on OCT imaging consistent with retinal dystrophy. **E**, **F** Pedigree and segregation analysis of variants in *MYH14* and *FBN2*. (+/+ variant present in homozygous state, ± variant present in heterozygous state, −/− wild type.)

Proband 52, a 48-year-old female with prelingual onset, bilateral, profound, steeply sloping high-frequency HI with type A tympanogram and bilateral reduced vision, nyctalopia, and photophobia starting at 5 years of age, showed two missense variants in *ADGRV1*, c.2261T>C p.(Val754Ala) and c.10878A>C p.(Lys3626Asn) (Table 1). Since two unaffected siblings were each heterozygous for either variant, the proband must be compound heterozygous, consistent with the diagnosis USH2C (Fig. 3B, C). The early onset and rapidly progressing vision loss (with light perception in the left eye and no light perception in the right eye), which is incompatible with USH2C, may be explained by an additional homozygous variant c.4919G>A p.(Arg1640Gln) in *ABCA4* (Table 1), which causes Stargardt disease (Simonelli et al. 2000). The most recent ocular examination revealed bilateral pseudophakic status, pale discs, atrophy and pigmentary changes in the macula, atrophy, bone spicules and pigment clumps in the retinal periphery (Fig. 3A). The variants segregated in a heterozygous state in two unaffected brothers (Fig. 3B, C).

In addition to a homozygous frameshift mutation in *USH2A*, c.236_239dup p.(Gln81Tyrfs*28), proband 58 presented with a medically actionable pathogenic variant, c.733_734del p.(Gly245Argfs*39) in *KCNQ1* (Table 1) (Amirian et al. 2018), which is associated with long QT syndrome 1 (OMIM 192500) and familial atrial fibrillation 3 (OMIM 607554).

### Non-USH syndromes causing combined HI and retinal degeneration

Nine of 59 probands with combined HI and retinal degeneration displayed clinical entities different from USH (Table 2). These included well-known syndromes that should be considered as part of the differential diagnosis of USH as well as unexpected inborn errors of metabolism. In many of the identified cases, the age-dependent manifestation of additional symptoms and the large clinical heterogeneity rendered a clinical diagnosis difficult or even impossible, hereby supporting the implementation of genetic testing.

**Table 2:** Non-Usher syndromes underlying combined hearing loss and retinal degeneration

ID	Gene	Variant	Protein	Zygosity^a^	Classification	References	Phenotype
39	*CEP78*	c.515T>G	p.(Ile172Arg)	Hom	Uncertain significance	Novel	Cone-rod dystrophy and hearing loss 1
40	*CEP78*	c.515T>G	p.(Ile172Arg)	Het	Uncertain significance	Novel	Cone-rod dystrophy and hearing loss 1
	*CEP78*	c.534del	p.(Lys179Argfs*10)	Het	Pathogenic	Namburi et al. (2016)	
46	*PEX26*	c.349C>A	p.(Pro117Thr)	Hom	Uncertain significance	Novel	Peroxisome biogenesis disorder 1C
47	*ALMS1*	c.6299C>A	p.(Ser2100*)	Hom	Pathogenic	Novel	Alström syndrome
48	*ALMS1*	c.7471_7472del	p.(Ser2491Thrfs*5)	Hom	Pathogenic	Novel	Alström syndrome
49	*ALMS1*	c.11410C>T	p.(Arg3804*)	Hom	Pathogenic	Liu et al. (2017)	Alström syndrome
50	*IDUA*	c.956C>T	p.(Ala319Val)	Hom	Likely pathogenic	Beesley et al. (2001)	Scheie syndrome (MPS1-S)
	*USH2A*	c.3045C>G	p.(His1015Gln)	Het	Pathogenic	Pierrache et al. (2016)	
	*ADGRV1*	c.1563del	p.(Pro522Leufs*18)	Het	Pathogenic	Novel	
51	*PDSS2*	c.702 + 1G>A^b^	p.?	Het	Likely pathogenic	Novel	Coenzyme Q10 deficiency primary 3
	*PDSS2*	c.488G>A	p.(Arg163His)	Het	Uncertain significance	Novel	
	*USH2A*	c.174T>A	p.(Cys58*)	Het	Pathogenic	Novel	
54	*ABCC6*	c.1171A>G	p.(Arg391Gly)	Hom	Uncertain significance	Chassaing et al. (2004)	Pseudoxanthoma elasticum

^a^Hom = homozygous; Het = heterozygous

^b^Testing with an in vitro splice assay showed exon skipping, r.631_702del p.(Val211_Lys234del)

**Fig. 4:**
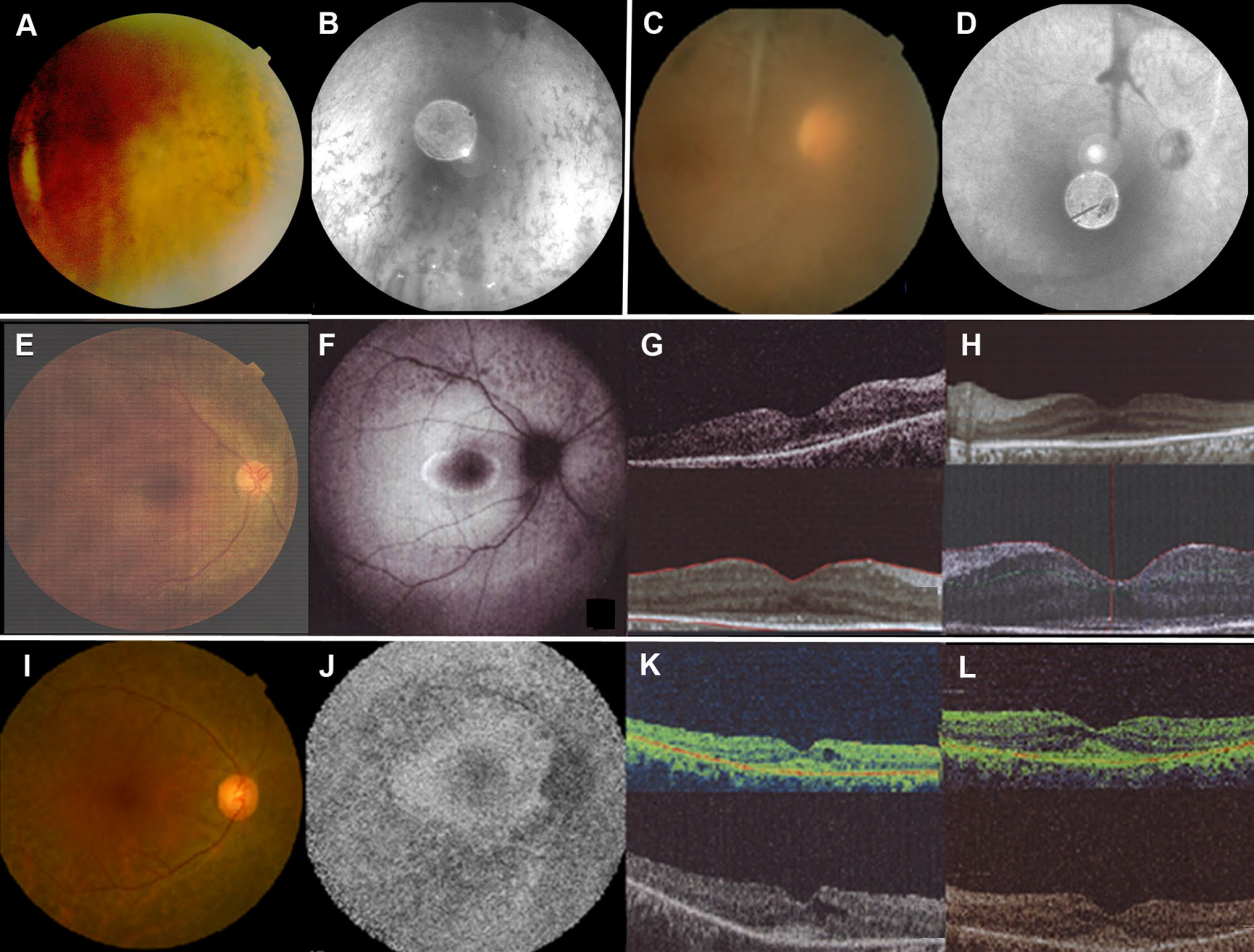
Retinal phenotype of selected probands with co-occurring HI and VI due to non-USH syndromic entities. **A**, **B** Fundus photography (FP) and fundus autofluorescence (FAF) in proband 49 with Alström syndrome (*ALSM1*) showing bone spicules in the retinal periphery (right and left eye) consistent with retinitis pigmentosa (RP). **C**, **D** FP and FAF of proband 46 with Heimler syndrome (*PEX26*) showing optic atrophy and pigment clumping despite reduced image quality due to cataracts (both eyes) consistent with RP. **E**–**H** FP, FAF, and optical coherence tomography (OCT) imaging of proband 39 with CRDHL1 (*CEP78*) showing decreased autofluorescence along the arcades on FAF imaging (right eye) and atrophy of the outer retina outside the center on OCT imaging (both eyes) consistent with RP. **I**–**L** FP, FAF, and OCT of proband 50 with Scheie syndrome (*IDUA*) showing decreased autofluorescence along the arcades on FAF imaging (right eye) and atrophy of the outer retina outside the center and mild cystoid macular edema on OCT imaging (both eyes) consistent with RP

It is noteworthy that three probands (47, 48, and 49) were endowed with different homozygous variants, c.6299C>A p.(Ser2100*), c.7471_7472del p.(Ser2491Thrfs*5), and c.11410C>T p.(Arg3804*), respectively, in *ALMS1*, causing Alström syndrome (OMIM 203800) (Table 2). This pleiotropic ciliopathy is associated with HI and VI, cardiomyopathy, endocrine, neurological, and hepatic symptoms (Rethanavelu et al. 2020). At the time of recruitment, our three Alström patients presented with photophobia, visual field constriction, posterior subcapsular cataracts, pigmentary retinopathy (Fig. 4A, B), and progressive sensorineural HI without vestibular dysfunction. Proband 47 suffered from bilateral severely reduced vision and bilateral profound steeply sloping high-frequency HI. Ophthalmological examinations revealed normal intraocular pressure, a pseudophakic status of the anterior segment, chalky white pale discs, severe macular atrophy (beaten bronze), diffuse bone spicule pigmentary changes, and mottling. At the time of examination, no clinical sign of cardiomyopathy was seen in our three Alström probands. Retrospectively, we learnt that proband 47 suffered from insulin-resistant diabetes and renal failure.

Two patients, 39 and 40, suffered from another ciliopathy, namely cone-rod dystrophy and hearing loss 1 (CRDHL1; OMIM 617236), due to homozygous or compound heterozygous variants in *CEP78*, respectively (Table 2). A possible founder mutation, c.515T>G p.(Ile172Arg) was identified. This variant has not been annotated in gnomAD, GME, LOVD, and HGMD. It was homozygous in a 32-year-old female (proband 39) who reported HI starting at the age of 5 years and bilateral reduced vision, photophobia and hemeralopia at 26 years. Clinical examination revealed pale discs, mild macular atrophy, diffuse retinal atrophy, and retinal pigment epithelium (RPE) mottling in both eyes (Fig. 4E–H). Kinetic and/or static visual field examination revealed a generalized depression with reduced mean deviation (− 3.344 dB; *p* < 0.01) with a central 10–2 threshold, consistent with RP. The ERG showed severe reduced amplitude in scotopic, photopic, and flicker responses.

A novel homozygous missense VUS in *PEX26*, c.349C>A p.(Pro117Thr) (Table 2) was found in a 12-year-old female (proband 46) with bilateral reduced vision, nyctalopia, bilateral subcapsular cataract, diffuse retinal atrophy with diffuse pigmentary changes in both eyes (Fig. 4C, D), and severely reduced amplitudes in scotopic and photopic ERG. This variant segregated in a heterozygous state in unaffected family members who were available for testing. Pathogenic variants in *PEX26* are associated with a wide spectrum of peroxisomal diseases, including Heimler syndrome, previously described in patients with concurrent HI and VI (Neuhaus et al. 2017). When the molecular diagnosis was communicated to the recruiting clinic, amelogenesis imperfecta in secondary teeth was retrospectively identified, which is in line with Heimler syndrome.

The homozygous likely pathogenic variant c.956C>T p.(Ala319Val) in *IDUA* in proband 50 (Table 2) has been previously reported in patients with Scheie syndrome (OMIM 607016), a mild form of mucopolysaccharidosis type I (Beesley et al. 2001). The 65-year-old female patient presented with diffuse RPE mottling, CME, generalized depression in her visual field exam (Fig. 4I–L), and postlingual HI. There were no other evident clinical findings indicative of Scheie syndrome.

The 29-year-old proband 51 with optic atrophy, retinal degeneration, and sensorineural HI was compound heterozygous for a missense variant c.488G>A p.(Arg163His) and a splice site variant c.702 + 1G>A p.? in *PDSS2* (Table 2) that has been associated with coenzyme Q10 deficiency (OMIM 614652). Compound heterozygosity of the respective VUS and likely pathogenic splice variant was confirmed by segregation analysis in unaffected family members. In vitro splice testing of the *PDSS2* c.702 + 1G>A mutant allele indicated skipping of exon 4 (257 bp), leading to an in-frame deletion, r.631_702del p.(Val211_Lys234del). The wild-type amplicon included the correctly spliced complete exon 4 sequence, showing as a 328 bp band (Supplementary Fig. 1).

The homozygous variant c.1171A>G p.(Arg391Gly) in *ABCC6* (Table 2) has been previously associated with pseudoxanthoma elasticum (Chassaing et al. 2004). Proband 54, a 31-year-old female, presented with progressive visual impairment, optic disc drusen, beaten bronze macular atrophy, diffuse RPE mottling, choroidal neovascularization, and postlingual sensorineural HI.

### Possible multi-locus genomic variation

Proband 44, a 42-year-old female, suffered from bilateral moderate high-frequency HI as well as late-manifesting (at the age of 38 years) severely reduced visual acuity, photophobia, nyctalopia, severe macular atrophy, diffuse pigmentary changes, and mottling with notable diminished central macular thickness (Fig. 3D). She displayed three novel missense variants; one, c.4732A>G p.(Lys1578Glu) in *MYH14*, and two, c.7355A>C p.(Glu2452Ala) and c.2507C>T p.(Thr836Met) in *FBN2* (Table 3). Since the unaffected brother did not exhibit *FBN2* variants (Fig. 3E, F) and other family members were not available, we do not know whether both variants reside on the same or on different parental alleles. Rare dominant variants in *FBN2* have been described in relationship with early onset macular degeneration (OMIM 616118) but also age-related macular degeneration (Ratnapriya et al. 2014). Variants in *MYH14* cause autosomal dominant HI (DFNA4A; OMIM 600652).

**Table 3:** Possibly solved, partially solved, and unsolved cases showing multi-locus variation

ID	Gene	Variant	Protein	Zygosity^a^	Classification	References	Phenotype
44^b^	*MYH14*	c.4732A>G	p.(Lys1578Glu)	Het	Uncertain significance	Novel	DFNA4A
	*FBN2*	c.7355A>C	p.(Glu2452Ala)	Het	Uncertain significance	Novel	Macular degeneration, early onset
	*FBN2*	c.2507C>T	p.(Thr836Met)	Het	Uncertain significance	Novel	
45^c^	*RHO*	c.659T>G	p.(Phe220Cys)	Het	Likely Pathogenic	Bunge et al (1993)	Autos. dominant or recessive RP4
	*MEFV*	c.2040G>C	p.(Met680Ile)	Het	Pathogenic	Tasliyurt et al. (2013)	Familial Mediterranean fever
53^c^	*PRPF8*	c.6462C>A	p.(His2154Gln)	Het	Uncertain significance	Novel	Autosomal dominant RP13
	*CACNA2D4*	c.2551 + 8C>T	p.?	Hom	Uncertain significance	Novel	Retinal cone dystrophy 4
57^d^	*USH2A*	c.5388T>A	p.(Cys1796*)	Het	Pathogenic	Novel	
59^d^	*MYO7A*	c.3750 + 7G>A	p.?	Het	Uncertain significance	ClinVar ID 43221	
	*CDH23*	c.5653C>T	p.(Arg1885Cys)	Het	Uncertain significance	ClinVar ID 522828	
	*G6PD*	c.1057C>T	p.(Pro353Ser)	Hemi	Pathogenic	Beutler et al. (1992)	Hemolytic anemia

^a^Hom = homozygous; Het = heterozygous; Hemi = hemizygous

^b^This case is considered as solved

^c^These cases are considered as partially solved, with variants explaining vision impairment

^d^These cases are considered as unsolved

Proband 45, a 40 year-old male, reported reduced vision and nyctalopia starting at 10 years of age and bilateral postlingual HI. He displayed macular pathology with pigmentary changes and bone spicules in the retinal periphery. He comes from a large consanguineous family. There are multiple members with dual sensory impairment and one brother with isolated HI. Both the proband and his deaf–blind youngest sister were endowed with a missense variant c.659T>G p.(Phe220Cys) in *RHO* (Table 3), that has been previously reported in patients with RP4 (OMIM 13731) (Bunge et al. 1993). Since the variant was also present in the hearing-impaired brother, reduced penetrance and/or variable expressivity must be assumed and the evidence for this variant is limited. Functional studies of RP-linked rhodopsin mutations have yielded conflicting results (Mallory et al. 2018; Lewis et al. 2020). The molecular basis of the HI in this family remains unknown. As an incidental finding, the proband displayed a common pathogenic variant in *MEFV*, that has been associated with familial Mediterranean fever and Behcet's disease (Tasliyurt et al. 2013).

In proband 53, a 13-year-old girl, we identified aa heterozygous variant, c.6462C>A p.(His2154Gln), in *PRPF8* (Table 3), which has been associated with RP13 (OMIM 600059), which is typically inherited as an autosomal dominant trait. In addition, she was endowed with a homozygous variant c.2551 + 8C>T in *CACN2D4* (Table 3), which has been associated with retinal cone dystrophy 4 (OMIM 610478). Variants in both genes may contribute to VI within the first year of life. A segregating variant underlying the HI has not been identified.

### Unsolved cases

In 3 of 59 probands (55, 57, and 59), we could not establish a molecular diagnosis for HI and/or VI. Interestingly, proband 57 exhibited a single pathogenic mutation, c.5388T>A p.(Cys1796*), in *USH2A* and proband 59 was heterozygous for two VUS, c.3750 + 7G>A p.? in *MYO7A* and c.5653C>T p.(Arg1885Cys) in *CDH23* (Table 3). Proband 59, a 47-year-old male with prelingual HI and VI since birth, was also endowed with a pathogenic variant, c.1057C>T p.(Pro353Ser) in *G6PD* (Beutler et al. 1992), which is associated with X-linked hemolytic anemia (OMIM 300908) in hemizygous males. Hyperbilirubinemia due to G6PD deficiency can induce neurological damage (kernicterus) in neonates and children, which may clinically manifest as auditory neuropathy spectrum disorder (Boskabadi et al. 2018).

## Discussion

As expected, most (44 of 59; 75%) of our deaf–blind patients were diagnosed with USH. Approximately half were USH1 and half USH2, with *MYO7A* (USH1B) and *USH2A* being the most prevalent genes. The relatively high proportion of *ADGRV1* (USH2C) (7 of 24; 29%) compared to USH2A (17 of 24; 71%) within the USH2 group may be explained by ethnicity of our cohort, mainly from Iran, which is an understudied population. In addition to USH, there is a wide variety of hereditary, non-hereditary and independent causes of HI and/or VI, which makes a correct diagnosis for clinicians and human geneticists challenging (Stiff et al. 2020).

Next generation sequencing (NGS) has greatly transformed the molecular diagnostics of both clinically and genetically highly heterogeneous neuro-sensory disorders, allowing rapid screening of large gene panels or the entire exome. Using WEA, we found genetic variants associated with combined HI and VI in 54 of 59 (92%) probands. Two probands (3%) were partially solved and only three (5%) remained without a molecular diagnosis explaining their phenotype. This is consistent with recent NGS screens of patients with combined HI and VI, which have yielded diagnostic efficiencies > 90% (Bonnet et al. 2016; Neuhaus et al. 2017; Jouret et al. 2019). This unusually high solve rate for a heterogeneous Mendelian disorder argues in favor of the notion that most genes underlying dual sensory loss have been identified. Our study also shows the utility of OCT for monitoring RP patients. The presence of macular edema, macular hole, epiretinal membrane, etc. has prognostic value and therapeutic implications.

The vast majority of variants underlying USH in our deaf–blind cohort were classified as pathogenic (44 of 63; 70%) or likely pathogenic (11 of 63; 18%) and most of them were already known (36 of 63; 57%). In contrast, the majority of presumably disease-causing variants in non-USH probands were VUS (10 of 17; 59%) and/or novel (12 of 17; 71%). This is not unexpected, considering that USH is one of the most extensively studied neuro-sensory disorders and updated comprehensive information on USH genes is available in variant data bases. Compared to USH, the data situation of the genes identified in probands with non-USH syndromes and multi-locus variation is relatively poor. Therefore, segregation analyses were performed in the families of the index probands to validate or discard variants.

### Non-Usher syndromes associated with combined hearing loss and retinal degeneration

Although USH is by far the most prevalent cause of deaf–blindness worldwide and also in our study, there are other syndromes that combine retinal dystrophy and HI with a number of additional symptoms. Five of our probands exhibited ciliopathies due to variants in *ALMS1* (Alström syndrome) and *CEP78* (CRDHL1). The absence of additional clinical symptoms in our three Alström patients may be explained by the variable and age-dependent expressivity of the phenotype. Only 17 and 19% of patients with Alström syndrome display intellectual disability and cardiomyopathy, respectively (Rethanavelu et al. 2020).

Heimler syndrome is a peroxisome disorder caused by biallelic variants in *PEX1*, *PEX6*, and *PEX26*. Proband 46 and several published cases with suspected USH presented biallelic mutations in *PEX1* and *PEX26* (Neuhaus et al. 2017; Diñeiro et al. 2020). The typical tooth (enamel) and nail abnormalities of Heimler syndrome were overlooked before molecular diagnosis. When assessed retrospectively, our proband presented mild amelogenesis imperfecta in secondary teeth.

Although it is known that patients with alpha-l-iduronidase deficiency (MPS1S or Scheie syndrome) can have HI and retinal degeneration, it was surprising that these were the cardinal symptoms leading to a molecular diagnosis (homozygous *IDUA* variant) in proband 50. This illustrates that mild phenotypic expression, in this case of a MPS1S, can complicate or delay diagnosis.

Another unexpected finding was compound heterozygosity of a likely pathogenic variant and a novel missense variant in *PDSS2* in proband 51. Coenzyme Q10 (CoQ_10_) deficiency is characterized by highly variable multi-systemic manifestations, ranging from fatal neonatal encephalopathy with hypotonia to isolated steroid-resistant nephrotic syndrome. The establishment of this molecular diagnosis in one of our probands with HI and VI is especially relevant, since high-dose oral CoQ_10_ supplementation can slow disease progression and even reverse some manifestations (Alcazar-Fabra et al. 2018).

Proband 54 was homozygous for a known variant in *ABCC6*, causing pseudoxantoma elasticum (OMIM 264800). This systemic elastic tissue disorder progressively affects the skin, forming yellowish papules that coalesce to form plaques until the skin becomes loose and redundant. Ophthalmological findings include angioid streaks, reticular macular dystrophy and speckled appearance of the macula. The clinical diagnosis is typically made in the second or third decade of life, when the skin and retinal symptoms are evident. Although HI has not been associated with pseudoxanthoma elasticum, it has been reported in patients with generalized arterial calcification of infancy (OMIM 624473), which is also caused by recessive variants in *ABCC6*.

Collectively, these results suggest that patients with dual sensory loss as the primary symptoms can suffer from a long list of syndromes (Stiff et al. 2020) that have HI and VI as part of their symptoms and with mild phenotypic expression or absence of additional symptoms that define the syndrome. In many monogenic disorders, the genotype is not predictive of the phenotype (Cooper et al. 2013). Variants that have been found in patients with highly variable phenotypic manifestations and apparently normal healthy individuals underscore the importance of variable expressivity and reduced penetrance.

### Blended phenotypes

Large WEA studies revealed dual molecular diagnoses in a considerable number of patients (Yang et al. 2014; Balci et al. 2017; Posey et al. 2017). Therefore, it is not surprising that three of our Usher probands exhibited additional variants in genes causing HI (*OTOG*, *TECTA*) or VI (*ABCA4*). One non-USH proband exhibited variants in different genes underlying HI (*MYH14*) and retinal or macular degeneration (*FBN2*). In two probands, we found variants segregating with VI but no variants in deafness genes. Altogether, in 6 (10%) of 59 probands dual sensory loss may represent a blended phenotype of variants in different genes for HI and/or VI.

In this context, it is noteworthy that although the vast majority of our patients come from consanguineous families, 20 of 54 (37%) solved cases are due to compound heterozygous variants in recessive genes. In 5 of 6 probands with likely multi-locus variation, dominant variants contributed to the phenotype. This accumulation of heterozygous variants in deaf–blind families may be at least partially related to phenotypic mating structure among individuals with HI and/or VI.

### Limitations

In probands with a single pathogenic *USH2A* variant (50, 51, and 57), partially solved (45 and 53), and unsolved cases (55, 57, and 59), CNVs and DIMs in *USH2A* were excluded by MLPA and Sanger sequencing. Similarly, CNVs in *PCDH15* were excluded by MLPA. In some probands (42, 43, 44, 45, and 58), genome-wide microarray screening revealed several CNVs; however, none were associated with HI and/or VI. Although we did not perform a comprehensive CNV analysis in all our probands, we can largely exclude a major contribution of CNVs to the etiopathogenesis of deaf–blindness in our cohort.

Despite diagnostic yields over 90%, some patients with dual sensory impairment remain without firm molecular diagnosis. This may be due to not yet discovered genes or variants in non-coding (intronic and regulatory) sequences, undetected CNVs and structural variants (i.e. inversions), or unannotated exons of known genes. Indeed, two of three unsolved patients displayed single pathogenic variants in USH genes, arguing in favor of the notion that we may have missed the mutation in the second allele. Although switching molecular diagnostics from WEA to whole-genome sequencing and improvement of diagnostic algorithms may overcome some of these problems, our capacity for interpretation variants outside the exome is still very limited.

### Benefits of improved molecular diagnostics

Many patients with neuro-sensory impairments benefit from an early molecular diagnosis, which may have important implications for disease management (i.e. tailoring optimum educational programs) and treatment (i.e. cochlear implantation, timely eye examinations and implementation of prophylactic or therapeutic measures to improve vision or slow the progression of retinal degeneration), prognosis (progressive or stable, development of additional symptoms), and family planning. Diagnosing syndromes in patients who are pre-symptomatic (for a given symptom) enables patients to consult respective specialists before symptoms manifest or progress. A variety of gene therapy approaches using adeno-associated viral vectors for gene delivery, antisense oligonucleotides or genome editing agents have already yielded promising results to prevent HI and retinal degeneration in murine USH models (Nagel-Wolfrum et al., 2014; Géléoc and El-Amraoui, 2020; Lentz et al. 2020), including clinical trials in patients related to mutations in exon 13 of *USH2A* (NCT03780257). Molecular diagnosis of the underlying defect is crucial to stratify patients for a growing number of successful ongoing clinical trials of ocular gene therapy, which represents a notable advancement to other inherited disorders (Cehajic-Kapetanovic et al. 2020).

Using state-of-the art clinical exome analysis, incidental or secondary findings unrelated to the primary reason for sequencing but of medical value for the proband, are identified in several percent of cases (Hart et al. 2019). The ACMG recommends returning highly penetrant pathogenic variants for a list of several dozen genes (Kalia et al. 2017; https://www.ncbi.nlm.nih.gov/clinvar/docs/acmg/). Important inclusion criteria are the possibility to confirm the associated phenotype, the availability of preventive measures and treatments, and that the mutation carriers can be asymptomatic for prolonged periods of time. Ideally, the patients should be alerted to this possibility before testing and have the chance to opt-out of receiving such unexpected findings. In this study we detected one medically actionable variant in *KCNQ1*, which is associated with long QT syndrome and has immediate implications for patient management. In addition, we found a pathogenic variant in *MEFV* which is associated with familial Mediterranean fever and a hemizygous variant in *G6PD*, which is associated with X-linked hemolytic anemia. Although not included in the list of actionable genes, these variants were considered as medically relevant and returned to the affected probands/families. Thus, altogether three (5%) probands in our cohort displayed secondary findings.

### Web resources

ClinVar, https://www.ncbi.nlm.nih.gov/clinvar.

Deafness Variation Database, https://deafnessvariationdatabase.org.

Hereditary Hearing Loss Homepage, https://hereditaryhearingloss.org.

Leiden Open Variation Database (LOVD), https://www.lovd.nl.

Moon Diploid, https://www.diploid.com/moon.

Mutation Distiller, https://www.mutationdistiller.org.

Primer 3, https://primer3.org/.

ACMG Recommendations for Reporting of Incidental Findings in Clinical Exome and Genome Sequencing, https://www.ncbi.nlm.nih.gov/clinvar/docs/acmg/.

Retinal Information Network, https://sph.uth.edu/retnet/disease.htm.

## Supplementary Information

Below is the link to the electronic supplementary material.

Supplementary file1 (PDF 395 KB)

## Data Availability

All data generated during this study are included in this article and supplementary information file. All variants have been entered into LOVD. Accession numbers are included in Supplementary Table 2.

## Declarations

### Animal experiments

Not applicable as no animal experiments were performed.
