# Supplementary material for: Unraveling the genetic complexities of combined retinal dystrophy and hearing impairment
Source: Hum Genet. 2021 Jun 20;141(3-4):785–803. doi: 10.1007/s00439-021-02303-1 (PMC9035000; doi:10.1007/s00439-021-02303-1)
Supplement: Supplementary file 1 — Supplementary file1 (PDF 395 KB) [file 439_2021_2303_MOESM1_ESM.pdf]

Supplementary Table 1 Clinical characteristics of index patients

ID[a]	Ethn.	Age	Sex	HLO	HLS	Aud.Shape	TYM	VIO	NL	RV	VA (LogMAR)	CME	RA	MA	PED	BPD	BC	SEI
1	Iran	65	F	Pre.	Prof.	Flat	A	27	X	X	OD 1.3; OS 0.6	X	X	X	X	X	X	
2	Iran	37	M	Pre.	n.a.	n.a.	n.a.	32	X	X	OD 1.0; OS 0.5	X	X	X		X	X	X
3	Iran	45	F	Pre.	n.a.	n.a.	n.a.	15	X	X	OD 1.0; OS 1.0							
4	Iran	35	F	Pre.	n.a.	n.a.	n.a.	Birth	X	X	OD 1.0; OS 2.0	X	X		X	X	X	X
5	Iran	46	M	Pre.	n.a.	n.a.	n.a.	20	X	X	OD 3.0; OS 2.0	X	X			X		
6	Iran	23	F	Pre.	Prof	Flat	A	25	X	X	OD 0.7; OS 1.0			X		X		
7	Iran	21	F	Pre.	n.a.	n.a.	n.a.	3	X	X	OD 0.7; OS 0.5	X	X	X		X		
8	Iran	5	F	Pre.	Prof.	Flat	n.a.	Birth	X	X	n.a.	X						
9	Iran	8	M	Pre.	Prof.	Flat	n.a.	4	X	X	OD 3.2; OS 2.0		X	X	X		X	
10	Iran	30	M	Pre.	n.a.	n.a.	A	20	X	X	OD 0.2; OS 0.4	X	X	X	X			X
11	Mex.	51	F	Pre.	n.a.	n.a.	n.a.	34	X	X	OD 3.2; OS 0.2		X		X	X	X	X
12	Iran.	30	M	Pre.	Prof.	Flat	n.a.	2	X	X	OD 2.0; OS 2.0			X		X		
13	Mex.	22	F	Pre.	n.a.	n.a.	n.a.	18	X	X	OD 0.2; OS 0.3	X			X	X		X
14	Mex.	8	M	Pre.	n.a.	n.a.	n.a.	8	X	X	OD 0.9; OS 0.9					X		X
15	Iran	37	F	Pre.	n.a.	n.a.	n.a.	15	X	X	OD 0.4; OS 0.4		X			X	X	
16	Iran	36	M	Pre.	n.a.	n.a.	n.a.	4	X	X	OD 0.7; OS 2.0		X	X	X	X		
17	Mex.	7	F	Pre.	Sev.	Flat	n.a.	n.a.	n.a.	n.a.	OD 1.0; OD 0.7	X			X			
18	Iran	38	F	Post.	n.a.	n.a.	n.a.	12	X	X	OD 0.3; OS 0.3	X	X	X		X	X	
21	Iran	28	F	n.a.	n.a.	n.a.	n.a.	19	X	X	OD 0.3; OS 0.2	X		X	X	X	X	
22	Iran	46	F	Post.	n.a.	n.a.	n.a.	35	X	X	OD 0.7; OS 2.0		X	X		X		
23	Iran	31	F	Post.	n.a.	n.a.	n.a.	12	X	X	OD 0.2; OS 0.4		X	X		X	X	
24	Iran	41	F	Post.	Sev.	Down slop.	n.a.	40	X	X	OD 0.7; OS 0.7		X	X		X	X	
25	Iran	26	M	Post.	Mod.	Steep. slop.	n.a.	16	X	X	OD 0.3; OS 0.3	X	X	X		X	X	
26	Iran	52	M	Pre.	n.a.	n.a.	n.a.	23	X	X	OD 0.5; OS 0.6	X	X	X		X	X	
27	Mex.	45	F	Post.	n.a.	n.a.	n.a.	23	X	X	OD 0.7; OS 0.6				X	X	X	
28	Mex.	29	M	Post.	n.a.	n.a.	n.a.	20	X	X	OD 0.5; OS 0.5	X	X	X	X	X	X	
29	Iran	30	F	n.a.	n.a.	n.a.	n.a.	18	X	X	OD 0.4; OS 0.3		X			X	X	

30	Iran	56	M	n.a.	n.a.	n.a.	n.a.	Birth	X	X	OD 3.0; OS 3.0		X	X	X	X	X	
31	Iran	45	F	Pre.	n.a.	n.a.	n.a.	Birth	X	X	OD 2.0; OS 2.0		X	X	X	X	X	
32	Iran	40	M	n.a.	n.a.	n.a.	n.a.	25	X	X	OD 0.2; OS 0.2		X	X				
34	Iran	44	M	Pre.	n.a.	n.a.	n.a.	22	X	X	OD 2.9; OS 2.9		X	X		X	X	
35	Iran	45	F	Post.	n.a.	n.a.	n.a.	7	X	X	OD 1.0; OS 2.0	X	X	X	X			
36	Iran	69	M	Post.	n.a.	n.a.	n.a.	51	X	X	OD 0.2; OS 0.2		X	X	X		X	
37	Iran	24	F	Pre.	n.a.	n.a.	n.a.	14	X	X	OD 0.3; OS 0.4	X	X	X		X	X	
38	Iran	66	M	Post.	n.a.	n.a.	n.a.	Birth	X	X	OD 0.5; OS 0.3		X	X		X	X	
39	Iran	32	F	Post.	n.a.	n.a.	n.a.	26	X	X	OD 0.8; OS 0.8		X	X		X	X	
41	Iran	24	M	n.a.	n.a.	n.a.	n.a.	14	X	X	OD 0.4; OS 0.4				X			
42	Iran	25	F	Pre.	Sev.	Flat	A	7	X	X	OD 0.3; OS 0.2		X	X	X	X		
43	Iran	45	F	Post.	n.a.	n.a.	n.a.	7	X	X	OD 1.0; OS 2.0		X	X	X	X		
44	Iran	42	F	Pre.	Mod	Flat	n.a.	38	X	X	OD 0.9; OS 2.0			X		X	X	
45	Iran	40	M	n.a.	n.a.	n.a.	n.a.	10	X	X	OD 0.3; OS 0.2							
46	Iran	20	F	Birth	n.a.	n.a.	n.a.	12	X	X	n.a.			X		X	X	
47	Iran	33	M	Post.	Sev	Steep. slop.	A	17	X	X	OD 3.2; OS 3.2		X	X		X	X	
48	Iran	35	F	Post.	n.a.	n.a.	n.a.	Birth	X	X	OD 2.9; OS 3.0		X	X		X	X	
49	Iran	32	M	Post.	n.a.	n.a.	n.a.	0.5	X	X	OD 2.9; OS 2.9		X	X	X	X	X	
50	Iran	65	F	Post.	n.a.	n.a.	n.a.	60	X		OD 0.3; OS 0.4			X		X		
52	Iran	48	F	Pre.	Sev	Steep. slop.	A	5	X	X	OD 3.2 OS 2.9							
53	Iran	13	F	Pre.	n.a.	n.a.	n.a.	0.75	X	X	OD 2.0; OS 2.9		X	X		X	X	
54	Iran	31	F	Post.	Mod.	Flat	n.a.	3	X	X	OD 3.2; OS 3.2			X				
55	Iran	62	M	Pre.	n.a.	n.a.	n.a.	Birth	X	X	OD 2.0; OS 2.0		X	X		X	X	
56	Mex.	7	F	n.a.	Prof.	Down slop.	n.a.	n.a.	n.a.	n.a.	OD 1.0; OS 0.9					X		
57	Iran	43	M	Pre.	n.a.	n.a.	n.a.	Birth	X	X	OD 1.0; OS 1.0	X	X	X			X	
58	Iran	17	M	Pre.	Mod.	Flat	A	10	X	X	OD 0.2; OS 0.1		X	X		X		
59	Iran	47	M	Pre.	n.a.	n.a.	n.a.	Birth	X	X	OD 0.2; OS 0.3		X				X	

[a] Patients 19, 20, 33, 40, and 51 with combined vision and hearing impairment were referred by experienced ophthalmologists to the Ophthalmic Research Center of Shahid Beheshti University for genetic diagnosis. However, no additional clinical information is available.

Abbreviations: ID: patient number; Ethn.: ethnicity (Iran = Iranian; Mex. = Mexican); Age (in years); Sex (M = male, F = female); HLO: hearing loss onset (Pre. = prelingual, Post. = postlingual); HLS: hearing loss severity (Mod. = moderate, Sev. = severe, Prof. = profound); Aud.Shape: audioprofile shape (Flat, Down sloping, Steeply sloping); TYM = tympanogram (A = normal middle ear functioning); VIO: visual impairment onset (in years); NL = nyctalopia; RV = reduced vision; VA = visual acuity (LogMAR = logarithm of the minimum angle of resolution, OD = right eye, OS = left eye); CME = cystic macular edema; RA = retinal atrophy; MA = macular atrophy; PED = pigment epithelium degeneration; BPD = bilateral pigment deposit; BC = bilateral cataracts; SEI = subjective equilibrium impairment; n.a. = information not available. For all dichotomous traits, X indicates presence of a given symptom and no X its absence.

Supplementary Table 2 Variants submitted to the Leiden Open Variation Database (LOVD)

ID	Gene	Variant c.	Variant p.	Zygosity[a]	Interpretation[b]	LOVD ID[c]
1	*MYO7A*	c.1969C>T	p.(Arg657Trp)	Hom	Pathogenic	00361770
2	*MYO7A*	c.73G>A	p.(Gly25Arg)	Hom	Pathogenic	00361775
3	*MYO7A*	c.5617C>T	p.(Arg1873Trp)	Het	Pathogenic	00361778
	MYO7A	c.2904G>T	p.(Glu968Asp)	Het	Pathogenic	
4	*MYO7A*	c.5573T>C	p.(Leu1858Pro)	Hom	Pathogenic	00361779
5	*MYO7A*	c.397dup	p.(His133Profs*7)	Hom	Pathogenic	00361780
6	*MYO7A*	c.2282+1G>C	p.?	Het	Pathogenic	00361781
	MYO7A	c.721C>T	p.(Arg241Cys)	Het	Pathogenic	
7	*MYO7A*	c.397dup	p.(His133Profs*7)	Het	Pathogenic	00361782
	MYO7A	c.4513G>T	p.(Glu1505*)	Het	Pathogenic	
8	*MYO7A*	c.487G>A	p.(Gly163Arg)	Hom	Pathogenic	00361783
9	*MYO7A*	c.3564_3571delinsA	p.(Tyr1188*)	Hom	Pathogenic	00361784
10	*MYO7A*	c.6204dup	p.(Ile2069Tyrfs*7)	Het	Pathogenic	00361785
	MYO7A	c.3564_3571delinsA	p.(Tyr1188*)	Het	Pathogenic	
11	*MYO7A*	c.722G>A	p.(Arg241His)	Het	Likely pathogenic	00374000
	MYO7A	c.1388A>G	p.(Gln463Arg)	Het	Likely pathogenic	
12	*MYO7A*	c.75_82del	p.(Ala26Glufs*13)	Hom	Pathogenic	00361786
13	*MYO7A*	c.5510T>C	p.(Leu1837Pro)	Het	Likely pathogenic	00374001
	MYO7A	c.487G>A	p.(Gly163Arg)	Het	Pathogenic	
14	*MYO7A*	c.496del	p.(Glu166Argfs*5)	Het	Pathogenic	00374002
	MYO7A	c.4117C>T	p.(Arg1373*)	Het	Pathogenic	
15	*MYO7A*	c.6228_6232del	p.(Asp2076Glufs*50)	Hom	Pathogenic	00361787
16	*MYO7A*	c.2914C>T	p.(Arg972*)	Hom	Pathogenic	00361788
18	*USH1C*	c.2191C>T	p.(Arg731Trp)	Het	VUS	00361789
	USH1C	c.658C>G	p.(Arg220Gly)	Het	VUS	
41	*CDH23*	c.7921G>A	p.(Asp2641Asn)	Hom	Likely pathogenic	00361790
17	*USH1G*	c.742C>T	p.(Gln248*)	Hom	Pathogenic	00362249
19	*USH2A*	c.4732C>T	p.(Arg1578Cys)	Hom	Likely pathogenic	00361791
20	*USH2A*	c.236_239dup	p.(Gln81Tyrfs*28)	Hom	Pathogenic	00361792
21	*USH2A*	c.1571C>T	p.(Ala524Val)	Het	Likely pathogenic	00361793
	USH2A	c.4628-2A>T	p.?	Het	Pathogenic	
22	*USH2A*	c.11955G>C	p.(Trp3985Cys)	Hom	Likely pathogenic	00361794
23	*USH2A*	c.12394del	p.(Leu4132Trpfs*35)	Hom	Pathogenic	00361795
24	*USH2A*	c.11357del	p.(Pro3786Leufs*6)	Hom	Pathogenic	00361796
25	*USH2A*	c.13510G>T	p.(Glu4504*)	Het	Pathogenic	00361797
	USH2A	c.13018G>C	p.(Gly4340Arg)	Het	Likely pathogenic	
26	*USH2A*	c.12067-2A>G	p.?	Hom	Pathogenic	00361798
27	*USH2A*	c.2512C>T	p.(Gln838*)	Het	Pathogenic	00374003
	USH2A	c.2299del	p.(Glu767Serfs*21)	Het	Pathogenic	
28	*USH2A*	c.5251_5267del	p.(Gly1751Leufs*2)	Het	Pathogenic	00374004
	USH2A	c.8141G>A	p.(Trp2714*)	Het	Pathogenic	
29	*USH2A*	c.5521G>A	p.(Gly1841Arg)	Het	Likely pathogenic	00361807
	USH2A	c.7915T>C	p.(Ser2639Pro)	Het	Likely pathogenic	
30	*USH2A*	c.8497dup	p.(Ser2833Lysfs*2)	Hom	Pathogenic	00361809
31	*USH2A*	c.12067-1G>C	p.?	Hom	Pathogenic	00362250
32	*USH2A*	c.8682-1G>A	p.?	Het	Pathogenic	00373532
	USH2A	c.2014C>T	p.(Gln672*)	Het	Pathogenic	

42	*USH2A*<br>*OTOG*<br>*PRPF31*<br>*ROM1*	c.11389+3A>T<br>c.7454del<br>c.632G>A<br>c.859C>T	p.?<br>p.(Arg2485Hisfs*77)<br>p.(Arg211Gln)<br>p.(Arg287Trp)	Hom<br>Hom<br>Het<br>Het	Pathogenic<br>Pathogenic<br>VUS<br>VUS	00362260
43	*USH2A*<br>*USH2A*<br>*TECTA*	c.5438C>A<br>c.7595-2144A>G<br>c.2572G>A	p.(Ser1813*)<br>p.? (DIM)[d]<br>p.(Asp858Asn)	Het<br>Het<br>Het	Pathogenic<br>Pathogenic<br>VUS	00361812
58	*USH2A*<br>*KCNQ1*	c.236_239dup<br>c.733_734del	p.(Gln81Tyrfs*28)<br>p.(Gly245Argfs*39)	Hom<br>Het	Pathogenic<br>Pathogenic	00373531
33	*ADGRV1*	c.9623+3A>C[b]	p.?	Hom	Likely pathogenic	00361814
34	*ADGRV1*	c.15736C>T	p.(Arg5246*)	Hom	Pathogenic	00361816
35	*ADGRV1*	c.4231del	p.(Ala1411Profs*6)	Hom	Pathogenic	00362251
36	*ADGRV1*<br>*ADGRV1*	c.4231del<br>c.10088_10091del	p.(Ala1411Profs*6)<br>p.(Val3363Aspfs*11)	Het<br>Het	Pathogenic<br>Pathogenic	00361817
38	*ADGRV1*	c.9512T>C	p.(Leu3171Ser)	Hom	VUS	00361821
52	*ADGRV1*<br>*ADGRV1*<br>*ABCA4*	c.2261T>C<br>c.10878A>C<br>c.4919G>A	p.(Val754Ala)<br>p.(Lys3626Asn)<br>p.(Arg1640Gln)	Het<br>Het<br>Hom	VUS<br>VUS<br>Pathogenic	00361827
56	*ADGRV1*<br>*ADGRV1*	c.5167C>G<br>c.14939T>C	p.(Pro1723Ala)<br>p.(Val4980Ala)	Het<br>Het	VUS<br>VUS	00362263
37	*CLRN1*<br>*CLRN1*	c.630del<br>c.625T>A	p.(Phe210Leufs*5)<br>p.(Phe209Ile)	Hom<br>Hom	Pathogenic<br>VUS	00361819
39	*CEP78*	c.515T>G	p.(Ile172Arg)	Hom	VUS	00361822
40	*CEP78*<br>*CEP78*	c.515T>G<br>c.534del	p.(Ile172Arg)<br>p.(Lys179Argfs*10)	Het<br>Het	VUS<br>Pathogenic	00361825
46	*PEX26*	c.349C>A	p.(Pro117Thr)	Hom	VUS	00361831
47	*ALMS1*	c.6299C>A	p.(Ser2100*)	Hom	Pathogenic	00361833
48	*ALMS1*	c.7471_7472del	p.(Ser2491Thrfs*5)	Hom	Pathogenic	00361835
49	*ALMS1*	c.11410C>T	p.(Arg3804*)	Hom	Pathogenic	00361837
50	*IDUA*<br>*USH2A*<br>*ADGRV1*	c.956C>T<br>c.3045C>G<br>c.1563del	p.(Ala319Val)<br>p.(His1015Gln)<br>p.(Pro522Leufs*18)	Hom<br>Het<br>Het	Likely pathogenic<br>Pathogenic<br>Pathogenic	00362252
51	*PDSS2*<br>*PDSS2*<br>*USH2A*	c.702+1G>A[b]<br>c.488G>A<br>c.174T>A	p.?<br>p.(Arg163His)<br>p.(Cys58*)	Het<br>Het<br>Het	Likely pathogenic<br>VUS<br>Pathogenic	00362261
54	*ABCC6*	c.1171A>G	p.(Arg391Gly)	Hom	VUS	00375267
44	*MYH14*<br>*FBN2*<br>*FBN2*	c.4732A>G<br>c.7355A>C<br>c.2507C>T	p.(Lys1578Glu)<br>p.(Glu2452Ala)<br>p.(Thr836Met)	Het<br>Het<br>Het	VUS<br>VUS<br>VUS	00362254
45	*RHO*<br>*MEFV*	c.659T>G<br>c.2040G>C	p.(Phe220Cys)<br>p.(Met680Ile)	Het<br>Het	Likely Pathogenic<br>Pathogenic	00362253
53	*PRPF8*<br>*CACNA2D4*	c.6462C>A<br>c.2551+8C>T	p.(His2154Gln)<br>p.?	Het<br>Hom	VUS<br>VUS	00362262
57	*USH2A*	c.5388T>A	p.(Cys1796*)	Het	Pathogenic	00375269
59	*MYO7A*<br>*CDH23*<br>*G6PD*	c.3750+7G>A<br>c.5653C>T<br>c.1057C>T	p.?<br>p.(Arg1885Cys)<br>p.(Pro353Ser)	Het<br>Het<br>Hemi	VUS<br>VUS<br>Pathogenic	00375266

[a] Hom = homozygous; Het = heterozygous; Hemi = hemizygous
[b] VUS = variant of uncertain significance
[c] LOVD ID refers to individual ID
[d] DIM = deep intronic mutation

ADGRV1 c.9623+3A>C

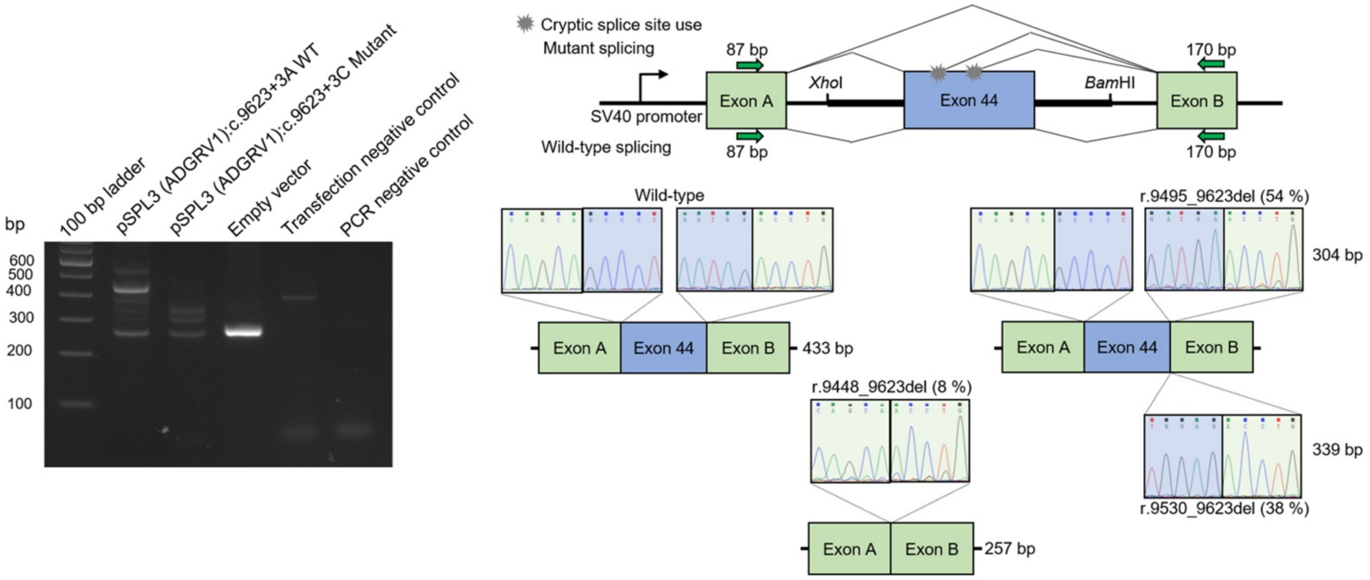

PDSS2 c.702+1G>A

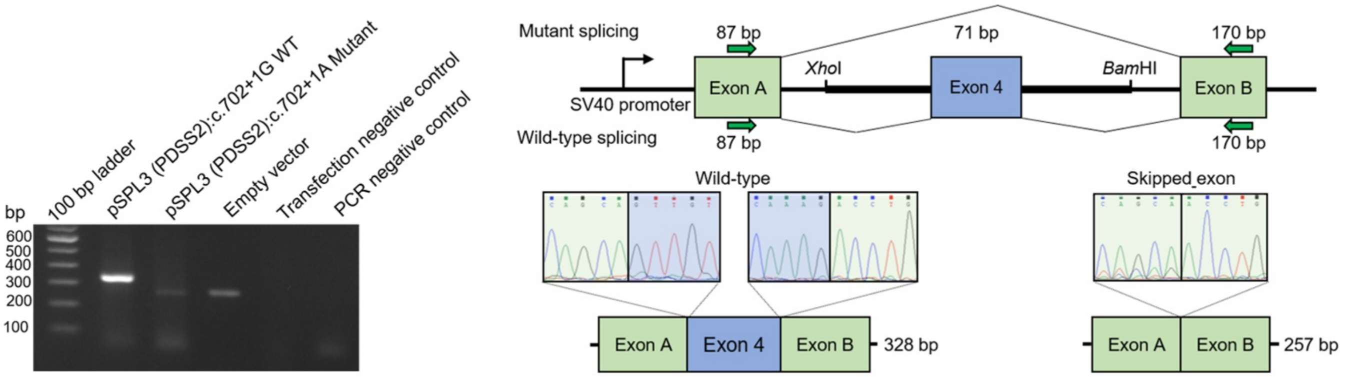

Supplementary figure 1 The upper panel shows the in vitro splice assay results for the *ADGRV1* c.9623+3A>C variant. Gel electrophoresis of the reverse-transcription polymerase chain reaction (RT-PCR) from the wild-type (WT) control, the *ADGRV1* c.9623+3C mutant allele, empty pSPL3 vector amplicons, and transfection negative and PCR negative controls. The vector construct illustrates the WT or mutant amplicons inserted between exons A and B of the pSPL3 vector with a splicing schematic of the c.9623+3C mutant (upper) and WT allele (lower). The WT (upper left sequencing panel) shows expected splicing. The mutant vector shows usage of two cryptic splice donor sites, yielding an in-frame deletion r.9495_9623del, p.(Tyr3166_Arg3208del), frameshift deletion r.9530_9623del, p.(Gly3177Glufs*5), and

skipping of exon 44 r.9448_9623del, p.(Ala3150Serfs*11). The relative fraction of abnormally spliced transcripts due to the variant are shown in percentages in parenthesis. The lower panel shows the in vitro splice assay results for the *PDSS2* c.702+1G>A variant. Gel electrophoresis of the RT-PCR from the WT control, the *PDSS2* c.702+1A mutant allele, and empty pSPL3 vector amplicons, as well as transfection negative and PCR negative controls. The vector construct of the in vitro splice assay illustrates the WT or mutant amplicons with a splicing schematic of the c.702+1A mutant (upper) and WT allele (lower). The WT (left sequencing panel) shows expected splicing. The mutant vector shows skipping of exon 4 (right sequencing panel), leading to an in-frame deletion r.631_702del, p.(Val211_Lys234del).
